# A Prototype for an Intelligent Water Management System for Household Use

**DOI:** 10.3390/s23094493

**Published:** 2023-05-05

**Authors:** Henrique Mamede, João Cortez Neves, José Martins, Ramiro Gonçalves, Frederico Branco

**Affiliations:** 1 CEG-UAb, Universidade Aberta, Rua da Escola Politécnica, 147, 1269-001 Lisboa, Portugal; 2 INESC TEC—Institute for Systems and Computer Engineering, Technology and Science, 4200-465 Porto, Portugal; 3 Inspiredblue Lda., Rua Francisco Grandela no. 2, 2500-487 Foz do Arelho, Portugal; 4 AquaValor—Centro de Valorização e Transferência de Tecnologia da Água, 5400-342 Chaves, Portugal; 5 Instituto Politécnico de Bragança, Campus de Santa Apolónia, 5300-253 Bragança, Portugal; 6 Department of Engineering, School of Sciences and Technology, Universidade de Trás-os-Montes e Alto Douro, 5000-801 Vila Real, Portugal

**Keywords:** intelligent water management system, Internet of Things, household water management, leak detection, water metering

## Abstract

Water scarcity is becoming an issue of more significant concern with a major impact on global sustainability. For it, new measures and approaches are urgently needed. Digital technologies and tools can play an essential role in improving the effectiveness and efficiency of current water management approaches. Therefore, a solution is proposed and validated, given the limited presence of models or technological architectures in the literature to support intelligent water management systems for domestic use. It is based on a layered architecture, fully designed to meet the needs of households and to do so through the adoption of technologies such as the Internet of Things and cloud computing. By developing a prototype and using it as a use case for testing purposes, we have concluded the positive impact of using such a solution. Considering this is a first contribution to overcome the problem, some issues will be addressed in a future work, namely, data and device security and energy and traffic optimisation issues, among several others.

## 1. Introduction

The wastage of water in households is a major concern that has adverse effects on both the environment and households. Consumer behaviour and pipeline infrastructure issues are the main causes of excessive water waste. Whether it is due to inadvertent actions by consumers or pipeline ruptures and problems, the issue of water wastage in domestic use is a reality. It is widely acknowledged that raising awareness about the problem is crucial in promoting more prudent use of this precious resource and reducing water waste [1].

Water consumption is likely to become more sustainable in the future as consumers become more aware of the problem of domestic water waste. Households will likely adopt more conscious and responsible water usage habits as water conservation education and campaigns become widespread. As a result, water waste caused by consumers’ unconscious actions will be reduced. Overall, the future of water consumption will see a shift toward more responsible water resource management and increased efforts to reduce water waste through individual behaviour changes and systemic infrastructure improvements [1]. Furthermore, more significant measures will be made to improve pipeline infrastructure to prevent water leaks and reduce water loss. Those efforts will necessitate investments in cutting-edge technology and materials that are more durable and resistant to wear and tear. While it is possible to make some progress toward more sustainable water consumption without technology, significant reductions in domestic water waste are only possible with technological advancements [2].

To minimise water wastage due to consumer behaviour, education and awareness campaigns are useful tools. However, to address pipeline infrastructure issues and prevent leaks, technology must also be employed. Advanced technologies, such as smart water meters, sensors, leak detection systems, and automated shutoff valves, can play a significant role in reducing water loss caused by pipeline issues. While education and awareness campaigns are crucial, technological advancements are also essential in significantly reducing domestic water waste. Information technologies such as the Internet of Things (IoT) can facilitate sustainable water consumption by providing real-time monitoring and management of water usage. The IoT can also generate valuable data and insights to help identify patterns of water waste and optimise water usage [3].

Taking this issue as a basis to state the problem under investigation, we can write the same as the need to respond simultaneously to intelligent water management in the domestic environment and the awareness of its use. Based on the Design Science Research methodology, we addressed the problem by conceptualising one architecture in an initial phase and then conceiving a cyber-physical artefact based on Internet-of-Things technologies, interconnecting several layers, physical and logical, from the hardware, communications, and software platform, in the cloud and a mobile environment, for each consumer. The software layer runs in cloud infrastructure, with Microsoft Azure chosen because of its IoT framework. The layer comprises three application groups: solution management, mobile user applications, and Azure IoT Hub. The management application controls overall device control and can detect water usage patterns for each user using machine learning. Users can interact with the control application via a web browser or mobile app, which includes a small local database for basic offline functionality. The solution employs the MQTT protocol, but other devices that support other protocols can be easily connected without requiring the solution to be recoded. Additionally, several other topics are intrinsically linked to the solution, specifically, those related to (1) data and device security, (2) traffic and communication optimisation between the devices and the cloud application, (3) the issue of the power required and the mechanisms that allow uninterrupted supply, and (4) the possibility of applying patches without the need to access the devices physically.

This article describes the process leading to the final solution, the validation, and its applicability.

This paper is organised as follows. In addition to this general outline section, Section 2 presents the relevance and work highlighting the need to find effective and efficient solutions to the global water consumption crisis, exposing the benefits of using IoT technology. Section 3 characterises the proposed architecture. In Section 4, the system developed to support and validate the proposed artefact is detailed. Finally, Section 5 presents research implications, the detected limitations, future work, and future considerations.

## 2. Water Management—Background Analysis

### 2.1. Water Leakage

Water leakage is a major issue worldwide, resulting in significant water losses, higher operational costs, and decreased water quality. Water leak detection and prevention at the cutting edge requires modern technology such as artificial intelligence, machine learning, and IoT sensors.

Several studies have assessed the efficacy of various technologies for water leak detection and prevention. Mashhadi et al. [4] found that machine-learning algorithms can effectively detect water leaks in large distribution networks. The work trained a machine learning model to identify leaks reliably, using data from pressure sensors and flow meters.

Another study looked into the usefulness of IoT-based water sensors for detecting leaks in residential structures. According to research [5], the sensors effectively detect leaks and might help reduce water losses and property damage.

A study by Rupiper et al. [6] revealed numerous best practices for reducing water leaks in extensive distribution networks. Standard pipe and valve maintenance, pressure control, and modern leak detection technology such as acoustic sensors and satellite images were among them.

Aside from these technologies, other investigations were carried out to study unusual approaches to detecting water leaks. According to El-Zahab and Zayed [7], thermal imaging can be used to identify water leaks in pipelines. Infrared cameras were utilised in the investigation to detect temperature abnormalities, which indicated the presence of leaks.

Despite all the advances, the problem of water leaks persists. Solutions are effectively lacking, and most of the research and development has been carried out at high-pressure distribution networks rather than at the level of domestic distribution networks or consumers’ properties.

### 2.2. Water Security

From a conceptual perspective, water scarcity refers to a tri-partied view of water composed of water scarcity, pollution, and water-related hazards and vulnerability issues [8].

While planet Earth is easily distinguished from the remaining planets since approximately 71% of its surface is water-covered, water scarcity is a real problem. According to the United Nations Food and Agriculture Organization and the World Economy Forum, it is one of the main challenges of the 21st century [9,10]. Considering the increase in water consumption registered in the last decade, it is critical to implement effective management of this natural resource [11].

Even though the current social uproar on water scarcity, a straightforward analysis of the existing literature allows us to easily perceive that this issue has been the focus of many researchers and research projects since the beginning of the 21st century [12,13,14]. Most of these propose innovative approaches to addressing the issue, whether from a consumption optimisation or a water management perspective. As a concept, water scarcity has been defined in multiple manners throughout the existing (i.e., scientific and grey) literature. Still, it can be consensually established as the condition where the available water fails to satisfy the current demand [15].

Water scarcity used to be the most pressing concern in water management; however, due to the intensifying effects of climate change, scientists and government entities have started shifting their attention towards other issues such as extreme flooding, over-exploitation of groundwater, and water pollution. The reason for this shift is the catastrophic social and economic effects that these issues can cause, and their impact on local, regional, and global sustainability [16].

Therefore, the essential point is that as a society, we must expand our water security-related perspectives and initiatives to ensure sustainable development in the long run. The primary approach to achieving this is taking stronger actions to tackle issues such as water shortages, water pollution, and water-related risks and vulnerabilities.

### 2.3. Water Management Digital Transition

As Yu et al. [17] and Sapkota et al. [18] argue, although water security is a global problem, it poses different challenges for urban and rural areas. The agri-food sector in rural areas frequently engages in unregulated water usage, intensifying the issue of water scarcity. In contrast, the urban context emphasises excessive consumption, pollution, and reducing water waste.

Hence, when addressing current water management and drawing on Hoffmann et al. [19] and Wilderer [20] arguments, there is a clear tendency for the rise of systems and solutions that are typically classified as grid solutions, non-grid solutions or hybrid solutions. Grids are essential components of today’s centralised systems, whose capital expenditure on pipes and sewers usually ranges from 70 to 80 per cent, creating effects of technological lock-in. Non-grid systems have plumbing inside individual structures and, on the grounds, no sewers or pipes connecting them. Hybrid systems incorporate both non-grid and (small) grid solutions into grid systems.

With the growing complexity of water management and the increasing adoption of digital technologies in societies and economies, the prospect of merging these two distinct contexts has become apparent. The adoption of digital technologies such as sensors, the Internet of Things (IoT), digital ecosystems, big data, and artificial intelligence in water management has the potential to promote the development of more efficient water monitoring, control, and forecasting solutions. It can also contribute to the sustainable collection, storage, and usage of freshwater [21,22,23].

Undoubtedly, urban and rural areas are becoming more digitised with the rapid growth and implementation of smart city and smart region concepts and the increasing connectivity of individuals and organisations to the Internet. Digital and smart technologies can enhance citizens’ quality of life, improve the effectiveness of local, regional, and national government entities, and promote sustainability in these territories [24,25].

Based on the active combination of IoT, sensors and artificial intelligence, smart water management solutions are currently understood in the existing literature as the future of water management and a powerful asset towards the continuous assurance of water security [26,27,28,29]. Several works can be found in the scientific literature, such as the ones enumerated in Table 1. Still, there are no concrete proposals of solutions and tools for the intelligent management of low-pressure water.

Many other past works follow the same line as those summarised in the previous table.

## 3. Intelligent Water Monitoring System Proposal

As the existing literature highlights, the advent of digital water management systems and the majority of the history associated with this type of system has targeted city or industrial-level solutions that should be adopted by local governments or significant water distribution or water consumption companies [39,40]. Despite this, given that most modern households are becoming more digital and “smart”, it is crucial to explore the development of new, more efficient, cost-effective, automated, and decision-support-focused systems. Such systems should be readily deployable in typical homes and not only aid in monitoring water consumption but also facilitate the acquisition of knowledge that promotes adopting sustainable water consumption practices within households [41,42].

### 3.1. Solution Requirements

By analysing all the arguments mentioned above, the following initial insights have been established:

- To the best of our knowledge, the existing literature needs to propose a water management solution to give the consumer instantaneous feedback on water consumption. Some existing solutions can collect data concerning water usage but not provide immediate feedback;
- Within the catalogue of analysed solutions and systems, no solution allowed for remote water flow control, as most of the researched solutions only addressed data collection concerning data usage;
- Although the proposal may seem promising, most of the revised models and architectures published in the existing literature have not been put into practice. Thus, whether they can effectively operate in a real-world setting remains to be demonstrated.

Many initiatives have been developed in the last ten years, but none with practical results and a strong focus on water usage in agriculture [43,44,45]. Numerous issues, such as the requirement for multiple layers and difficulties with technology integration, have not been sufficiently addressed. Additionally, there seems to be some confusion about where to put the control: on high or low pressure. We also found some technology-related problems at this level, but the focus is on high pressure [16,36,46]. Recently, some companies started applying technology to domestic water consumption metering, based on Low-Power Wide-Area Network (LPWAN), communication protocols such as LoRa, Narrowband–IoT (NB–IoT), and other licensed protocols such as SigFox or even supported by PAN/Mesh Network technology such as ZigBee. Mainly to collect consumption information or sensors able to detect a sudden leak at home, providing only an alert (i.e., home automation systems).

In conclusion, no architecture and the respective implementation for an intelligent water management system was found, nor a feedback model that could lead to the creation of control and, at the same time, able to provide a more significant consumer awareness. The conclusion supported the writing of the research question about how to let individuals control the water they use while creating awareness of that consumption.

Besides the research question, two objectives have been defined: (1) How do we reduce house water waste? (2) How do we create a more rational use of the resource?

Focusing on the answer to the research question while looking to reach the stated objectives, we started by defining a certain number of requirements for our proposal, namely:

- Every piece of the solution must be as standard as possible;
- The solution will use only open and non-proprietary communication protocols;
- The solution must provide the ability to put the data/information in each user’s hand;
- The search for the solution must consider the transformation of the traditional water meter into an intelligent device;
- We may propose additional devices to complement the meter activity.

In Table 2, we emphasise the requirements of the system and their impact on water management.

### 3.2. Solution Architecture

The Internet of Things aspires to create a pervasive computing environment where ordinary objects allow interoperability to achieve a shared purpose [47]. It may be considered an interconnection between the physical world and the Internet. We can also control our physical environment using these applications.

**Figure 1:**
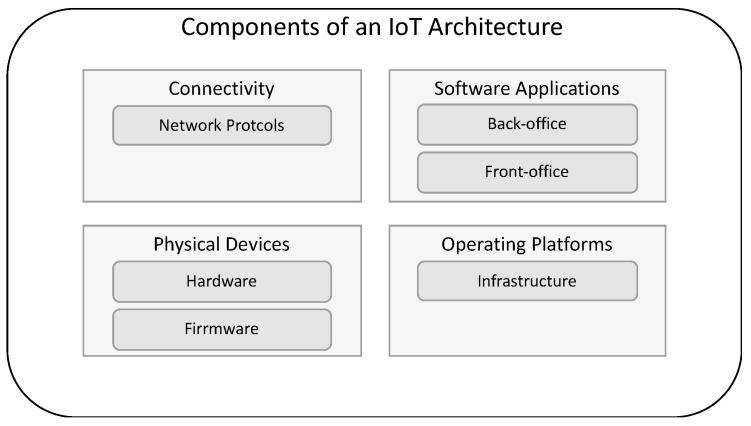
Components on a generic IoT architecture.

Several authors have long discussed general architecture [47,48,49,50]. Based on the various generic architectures in the literature, we designed one that would fit our requirements. It is illustrated in Figure 1.

**Figure 2:**
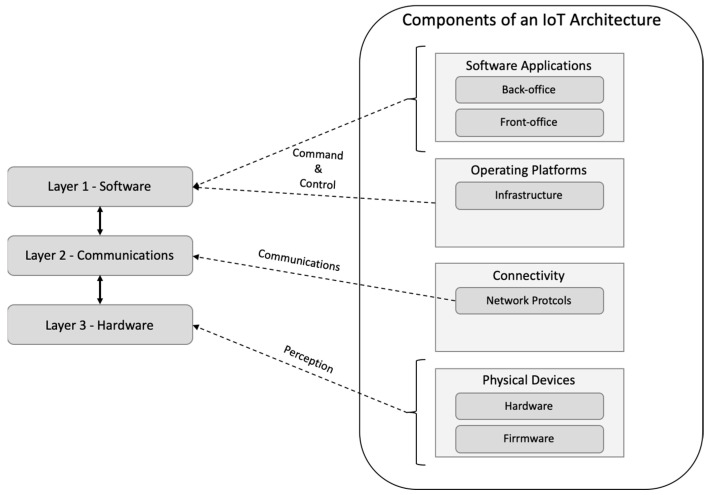
Components of a generic IoT architecture.

Considering the components detailed in the previous figure, we tried to simplify such a general architecture. This effort took us to a layered approach as the final architecture, as illustrated in Figure 2. From the requirements, we started to divide the possible solution into several different layers, approaching the partial solution on each layer and dealing with the integration later. Internet-of-Things solutions were found as the most suitable due to their characteristics [51,52]. Figure 2 depicts, on the left side, the layers typically found on any cyber-physical solution based on Internet-of-Things technologies.

This layered approach allows for an independent architecture and implementation within each layer, considering their communication processes. Additionally, it is advantageous that we can easily change or update the technology in one layer without affecting the others.

We based our work on those three layers:

- Layer 1—Software, considering the requirements for an end-user solution able to fulfil the objectives and comply with the requirements;
- Layer 2—Communications, considering the usage of non-proprietary protocols and the need to support different approaches, thinking on a global perspective of later broad implementation;
- Layer 3—Hardware, defining and engineering the needed hardware to support the solution’s requirements.

**Figure 3:**
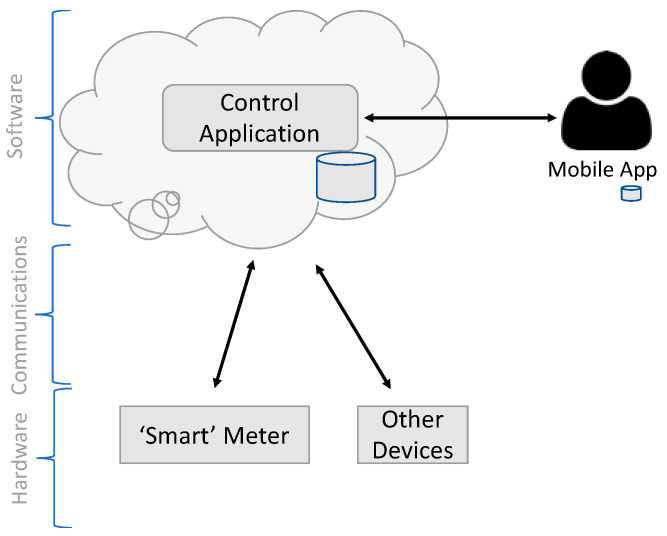
Layered solution architecture.

The final architecture is represented in Figure 3.

Based on this layered approach, we will detail every aspect of the architecture.

#### Software Layer

The software layer is executed in cloud infrastructure, and we chose Microsoft Azure due to many accelerators already available through its IoT framework [53]. So, this layer can be divided into three groups of applications: (i) application developed for the management of the solution, filling the general requirements previously stated; (ii) mobile application for the user; (iii) Azure IoT Hub, supporting the interface through message queue management, supporting MQTT, AMQP, COAP, and, even, webhooks, relieving us from the effort of coding and keep those protocol implementations updated [54]. Our solution uses MQTT protocol, but with our environment, connecting the future new devices supporting other protocols will be easy and without recoding any part of the developed solution.

**Figure 4:**
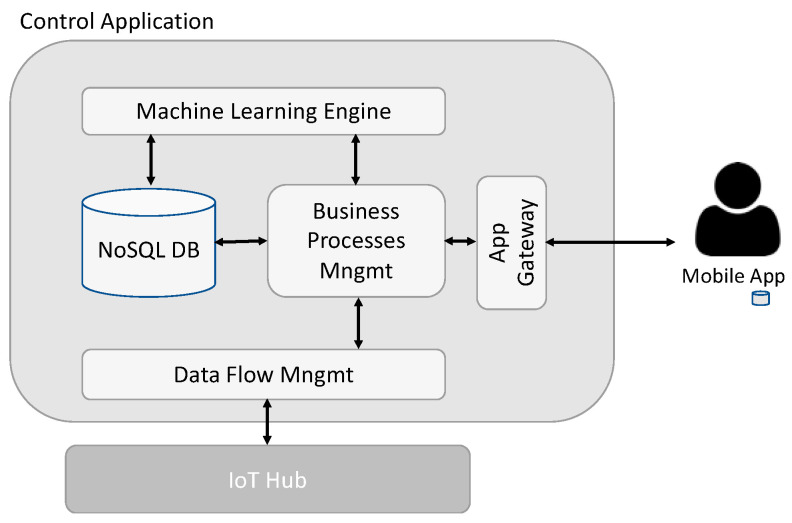
Software layer components.

The application developed to manage the solution is responsible for the global control of all devices, providing all the functionalities to handle them (intelligent water meter and other devices). It can receive all incoming data from the devices, with information collected by all the sensors, and provide commands to the same devices. It also allows the provision of devices and customer accounts, linking both. As seen in Figure 4, this control application has a machine learning engine that, acting on collected data, can detect the water usage patterns for each user, classifying them on a specific profile.

The user can interact directly with the control application through a web browser or use a mobile app developed for data consumption and alert management. This mobile app has a small local database to provide fundamental offline analysis and deal with performance when there are already many data collected for a user. From the mobile app, the user can analyse its data and interact with devices sending commands, e.g., water shutoff, total or partial.

From an implementation point of view, this control application uses a specific IoT Hub from Microsoft, provided as an Azure service, that supports all the different communication processes, stripping and isolating the application from implementation details at that level. The IoT Hub delivers data directly into a module that deals with data flow or receives data (i.e., commands) to be sent to a specific device.

This option has been taken because it easily supports different data types (e.g., structured and non-structured) and provides low transaction latency. The business processes implemented and supported by a NoSQL Database, also from Azure, deal with the data. The data is also accessed via the machine learning engine, which looks for patterns and specific alerts from sensors and makes decisions about actions, transforms them into commands, and sent to devices.

There is also an application gateway to integrate with the mobile app, which can run on iOS or Android.

This modular structure approach allows us to change or improve some application pieces without impacting others. At the same time, the used Azure services remove the complexity of dealing with some aspects that changes through time, namely communication processes.

### 3.3. Communications Layer

The devices need communication. Otherwise, they would not be able to send the sensor data to the control application, nor would the control application be able to send action commands.

However, we soon understood that we needed to solve problems such as frequent loss of connectivity and fast battery drainage when there are no electricity and coverage problems. We started by engineering a solution based only on Wi-Fi standards (i.e., IEEE 802.11). From here, we decided to also implement support for GSM and LoRaWAN.

GSM is intended to be used when there is no other communication channel since it imposes a particular cost in the communication process and battery drainage but gives us an almost global-wide coverage area.

LoRaWAN is an LPWAN protocol (Low-Power Wide-Area Network), non-proprietary and working on open frequencies, developed precisely to support Internet-of-Things (IoT) communications [55,56]. Since there is growing coverage of LoRaWAN worldwide, it seemed to be a good decision [57]. We used a gateway from a vendor, which also provides its own Network Server.

A device supports only one of those protocols for cost reasons, so the end user must decide on the installation.

### 3.4. Hardware Layer

The hardware devices are not only sensors; we also use actuators. The water meter can efficiently measure the water flow due to high-resolution ultrasound sensors. It can be mounted on any position and measure the water flow in both directions. Water consumption also measures temperature, water pressure, and time.

**Figure 5:**
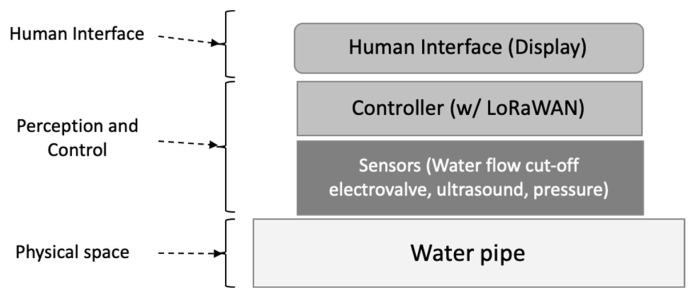
Hardware device schematics.

This device can receive commands from the control application to close the water supply (total or partially). From the end user’s point of view, there could be a feeling that the water meter can interact with other devices, such as our leak detector, but it is performed through the control application. In the same way, the water meter generates alerts and information that is sent to the control application. The actual “intelligence” of the device is provided via the control application, previously described, which executes in the cloud. A controller that includes LoRaWAN was chosen. The final schematic for the water meter device is illustrated in Figure 5.

## 4. Solution at Work

We can combine the solution architecture with the initial requirements to ensure they are covered. Table 3 shows this verification.

**Figure 6:**
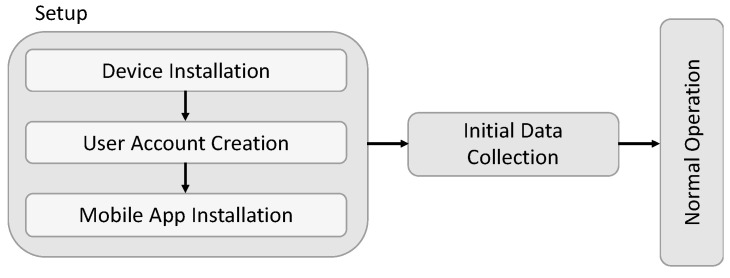
Solution Setup.

This solution is different from a “plug-and-play” solution. It requires specific initial steps to allow regular operation, as represented in Figure 6.

First, there is a need for an initial setup, which is composed of three actions: (1) The intelligent water meter device is properly installed, put in place, and working; (2) The customer installs the mobile app on his smartphone; (3) The customer account has been created in the control application, and the device has been associated with it.

After this setup, the data incoming from the meter starts to be collected for a whole week (i.e., seven days) until it starts the regular operation.

The initial data collection is delivered to the Machine Learning engine through the Control Application. The result is profiling this installation so that the software can detect a leak from regular water usage. Ultimately, the system can show the different profiles for all users or by city. Based on this data, the system uses gamification to create virtual rewards for users when their usage profile registers a lower water consumption. The virtual rewards are delivered through the mobile app.

There is also an “away mode”, allowing the users to define a period of non-usage of water. This is particularly important during holidays or weekends.

When the meter detects that in a short time (i.e., one minute), there is a consumption equal to or bigger than 100 litres, it generates a “slow leak alert”. That is propagated to the user’s mobile app; any other detection, if greater, generates a “leak alert”, and the Control Application can be configured to automatically close the water after some time, in case the user does make any action. Of course, we want to note this because usage is profiled so that the meter can distinguish a leak from a regular use for that installation.

When unexpectedly water starts to flow for some time (i.e., it can be seconds), it starts by generating an alert informing that the water will be closed unless the user acts in the mobile app. If there is no action, the meter shuts off the water, alerting the customer through email and SMS.

The water meter communicates every two hours, providing all the collected data in regular operation. If a leak is detected, the water meter immediately communicates the event.

Note that, complementing the action of the water meter, we developed a small device for leak detection that should be disposed of where there is a more significant probability of a water leak. These devices operate independently, and they have communication capabilities. Placing examples are below washing machines and in the back of toilets.

If the leak detector device detects the presence of water, it will enquire the meter in that installation, and if there is water passing by, then a leak is detected. The customer is alerted via email and SMS. The water supply is automatically shut off.

Of course, it is not the leak detector that inquires about the meter. The leak detector will send an alert to the control application, which, in turn, will send a command requesting the appropriate information from the meter in that installation.

Acting like this, if someone accidentally spills water in the device, it can differentiate from an actual water leak.

**Figure 7:**
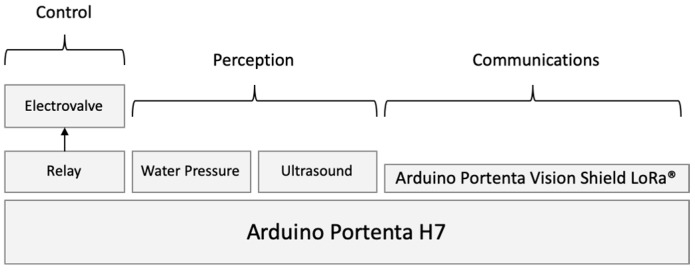
Schematic solution for used components.

Two prototypes have been built. Those prototypes have been installed on two properties in different regions, about 100 km apart. The schematic solution and the used components are illustrated in Figure 7.

The installation went smoothly and as expected, although the intervention of a plumber was necessary for both situations to ensure the correct installation of the physical device. The installation went smoothly and as expected. A gateway from a vendor was used with no coverage problems due to the distance between the physical device and the gateway (i.e., 15 m in one case; 25 in the other).

**Figure 8:**
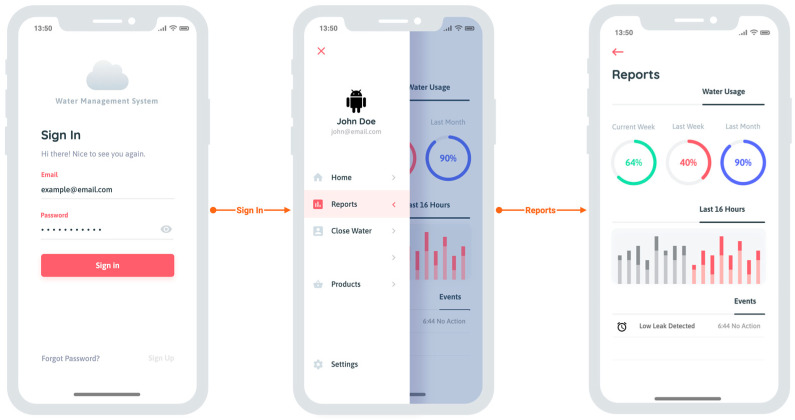
Mobile app developed to give the end user information and control over its installation.

The collection of profiling data went as expected. After seven days of data collection and activating all the solution’s functionalities, only one false-positive was identified after two months of use. This was due to the need for an intervention in the garden’s irrigation system, whose test led to the system’s closure due to detecting a large-scale leak and subsequent alert to the user, the property owner. Figure 8 illustrates the mobile app developed to give the end user information and control over its installation.

Table 4 illustrates the collected data for the initial four months of using the solution. Starting from an average (i.e., for the last 12 months) of water consumption, we can easily understand the importance of having the users know how much water they spend just a few moments after the event.

Interestingly, a 17% reduction was observed in the water consumption of the properties with the solution installed. When we analysed the reasons for this reduction, we realised that it was caused from allowing users to see the water consumption values immediately after events such as, e.g., a bath or washing the car.

We consider this result unexpected insofar as we anticipated that something in this context might happen, but never at such a significant level of consumption reduction.

On the other hand, specific concerns have been raised that necessitate careful consideration and further refinement. These concerns comprise the following:

- The quantity of water that flows back into the network from home infrastructure when a tap is opened and closed is not accurately measured;
- A mechanism must be developed to update the firmware of meters supported in the communications network to enable the application of corrections and updates without the need to dismantle and send them back to the factory.

## 5. Conclusions

### 5.1. Research Implications

The proposed intelligent water management system represents, from our perspective, a valuable novelty as it not only advocates a more agile and efficient water management approach but also comprises a layered architecture composed of three distinct layers. Each has a very individual and outlined a set of functions, but all-embracing the necessity of being interoperable.

Hence, from a theoretical perspective, the establishment of novel layered architecture, supported by a very up-to-date literature background, that perceives the relevance of having a software layer aligned with the current Software-as-a-Service and Infrastructure-as-a-Service paradigms, combined with a communication layer that represents the necessity for ensuring wireless and low-power communications between remote and central nodes, and a hardware layer that mimics the actual electronic and physical elements of the proposed water management system and their integration abilities, represents a valuable contribution for current and future researchers in the field of intelligent water management.

Concerning the more practical contributions of our research, they are related to the practical implementation of the proposed artefact. This new knowledge created during the referred implementation process, alongside the technical and functional details associated with each of the system layers, can be used by existing companies to develop their water management solutions further or as the baseline for the development of new commercial-graded solutions that they can market. At the same time, a contribution was made to developing solutions capable of managing and minimising water consumption, an increasingly scarce resource, through individual awareness. Table 2 and Table 3 show a list of the requirements and the impact of each on water management.

### 5.2. Limitations and Future Research

The main limitation is that we are still running a small number of installations (i.e., 15). Despite this, they are spread, with some more than 200 km from the others, so they are settled in regions with very different characteristics concerning water availability. We need to understand if there is any correlation between those characteristics and the usage of our system.

Our future efforts aim to improve the functionality of the intelligent water management system and make it compatible with older and conventional water meters. We will prioritise enhancing the security of data and devices, optimising traffic and communication between devices and the cloud application, addressing power-related issues, and implementing mechanisms for uninterrupted supply.

Additionally, we plan to develop a mechanism for applying patches without the need for physical device access. As the number of devices increases, the amount of data collected by all sensors will also increase. Therefore, we will explore edge computing models to keep the central solution manageable.

### 5.3. Final Considerations

We started with the question about letting individuals control their water while creating awareness of that consumption.

After researching the state of the art, we found no solution to help provide a proper answer to that question.

From here, we engineered a solution based on Internet-of-Things technologies, with a multi-level architecture, able to support us in the search for a proper answer. With the solution we developed, now every user can avoid water wastage, giving control to everyone (sometimes, taking control of the situation in an emergency). Additionally, we collect, process, and deliver all the data related to that process. At the same time, we provided an architecture able to support any IoT device independently of the message system it uses.

That means users can now create awareness about their water consumption. Our data shows that everyone tends to reduce the water consumed as soon as the first month after installing the system.

As the main benefits of using this solution, we find the following: remote access to each water installation; comparisons and insights; gamification; real water saving (17%); conservation of pipes infrastructure on each home; leak detection; alerts; real-time measurement; wireless communication; reports (i.e., real-time and analytics); dashboard for remote access; forecast/insight.

## Figures and Tables

**Table 1:** Research on smart water management.

Reference	Comments
[30,31,32,33]	Do not propose real working systems, but a data-based solution for consumption prediction over a certain number of households and for decision-making on water distribution.
[34,35]	Propose one IoT solution at the distribution level (high-pressure), not the consumer level (low-pressure).
[36]	Discussion about the lack of industry standards for water management
[37]	Water distribution management in a specific situation where every home possesses a tank.
[38]	Smart water management pillars in the context of smart cities.

**Table 2:** Impact of requirements on water management.

Requirement	Impact on Water Management
Every piece of the solution must be as standard as possible	Using standard elements will impact the time to market any developed solution and the time to repair when needed. In this way, any problem caused by failure or lack of the device is minimised.
The solution will use only open and non-proprietary communication protocols	Using non-proprietary protocols will facilitate network coverage and make the communication process cheaper while minimising the risks of failure of connections and, consequently, the exchange of information and commands between devices and the cloud application.
The solution must provide the ability to put the data/information in the hand of each user	Suppose each user, at any given moment, can understand their consumption and have a solution that minimises losses through leakages and other problems of a similar nature. In that case, we will have the individual capacity to manage water.
The search for the solution must take into consideration the transformation of the traditional water meter into an intelligent device	A smart meter will allow each user to carry out individual water management, providing them with data for decision making and the tools capable of independently acting in an emergency.
We may propose additional devices to complement the meter activity	Additional devices allow greater granularity of information to the consumer, superior installation protection, and greater precision.

**Table 3:** Checking requirements and the solution.

Requirement	Addressed by
Every piece of the solution must be as standard as possible.	Software layer based on Microsoft IoT Framework; communications supported in LoRaWAN; hardware uses a standard controller and devices from the market.
The solution will use only open and non-proprietary communication protocols.	Communications supported in LoRaWAN.
The solution must provide the ability to put the data/information in each user’s hand.	Mobile application that connects to Control Application, allowing the user to consume data and interact with devices.
The search for our solution must take into consideration the transformation of the traditional water meter into an intelligent device.	The device can shut down the water by itself; the control application provides data and recommendations for better water usage.
We may propose additional devices to complement the meter activity.	A small device has been developed for local leak detection, completing the solution.

**Table 4:** Collected data.

			Water Consumption (in Litres)				
	Characteristics	Number of Persons	Last 12 Months (Monthly Average)	Month 1	Month 2	Month 3	Month 4
Property 1	Villa w/garden	3	17,600	15,710	14,860	14,310	13,770
Property 2	Villa w/garden	4	23,200	20,265	19,678	18,678	18,045

## Data Availability

The data presented in this study are available upon request from the corresponding author. The data are not publicly available due to institutional indications.
